# Investigating 2-D and 3-D Proximal Remote Sensing Techniques for Vineyard Yield Estimation

**DOI:** 10.3390/s19173652

**Published:** 2019-08-22

**Authors:** Chris Hacking, Nitesh Poona, Nicola Manzan, Carlos Poblete-Echeverría

**Affiliations:** 1 Department of Geography and Environmental Studies, Stellenbosch University, Private Bag X1, Matieland 7602, South Africa; 2 Dipartimento di Scienze AgroAlimentari, Ambientali e Animali, University of Udine, Via delle Scienze 208, 33100 Udine, Italy; 3 Department of Viticulture and Oenology, Faculty of AgriSciences, Stellenbosch University, Private Bag X1, Matieland 7602, South Africa

**Keywords:** Kinect sensor, RGB, RGB-D, image segmentation, colour thresholding, bunch area, bunch volume, point cloud, mesh, surface reconstruction

## Abstract

Vineyard yield estimation provides the winegrower with insightful information regarding the expected yield, facilitating managerial decisions to achieve maximum quantity and quality and assisting the winery with logistics. The use of proximal remote sensing technology and techniques for yield estimation has produced limited success within viticulture. In this study, 2-D RGB and 3-D RGB-D (Kinect sensor) imagery were investigated for yield estimation in a vertical shoot positioned (VSP) vineyard. Three experiments were implemented, including two measurement levels and two canopy treatments. The RGB imagery (bunch- and plant-level) underwent image segmentation before the fruit area was estimated using a calibrated pixel area. RGB-D imagery captured at bunch-level (mesh) and plant-level (point cloud) was reconstructed for fruit volume estimation. The RGB and RGB-D measurements utilised cross-validation to determine fruit mass, which was subsequently used for yield estimation. Experiment one’s (laboratory conditions) bunch-level results achieved a high yield estimation agreement with RGB-D imagery (r^2^ = 0.950), which outperformed RGB imagery (r^2^ = 0.889). Both RGB and RGB-D performed similarly in experiment two (bunch-level), while RGB outperformed RGB-D in experiment three (plant-level). The RGB-D sensor (Kinect) is suited to ideal laboratory conditions, while the robust RGB methodology is suitable for both laboratory and in-situ yield estimation.

## 1. Introduction

Modern-day viticulture has seen an increase in the use of robust scientific methods combined with new technologies to improve overall production [1]. Precision farming, a direct result of the modernisation in agriculture, can be discipline-specific—i.e., specific to horticulture or viticulture. Precision viticulture aims to effectively manage production inputs to improve yield and grape quality while reducing the environmental impact of farming [2]. The use of remote sensing technology and techniques in precision viticulture allows variability to be monitored at vineyard level, per individual block or on a vine basis. Aspects such as vine shape, size and vigour can be observed, providing more accurate yield and fruit quality information [3].

Yield estimation provides information to the winegrower that can be used to manage the vineyard, optimising quality and yield [4]. Awareness of the estimated yield allows the vineyard manager to manipulate the vines to obtain the desired grape characteristics, and provides an effective plan for use during the winemaking process [5]. Accurate yield forecasting assists with logistical planning, both during and after the harvest; for example, what volume will be harvested, where the grapes will be stored, and an expected market price [5].

Wolpert and Vilas [6] outlined a two-step method for vineyard yield estimation. The start of the process determines the number of bunches situated on individual vines early in the season. Subsequent determination of bunch weights occurs at vèraison. Unfortunately, the two-step method is labour-intensive, error-prone and destructive in the estimation process. Additionally, De la Fuente et al. [7] presented yield prediction models using destructive, manually collected data between fruit-set and vèraison, aligning with the more ‘classical’ two-step method. To overcome the limitations of the manual methods, modern techniques have employed sensors attached to automatic harvesters to monitor yield during the harvesting process [3]. Yield estimation before harvest is becoming possible, with increasing accuracies when making use of non-invasive proximal remote sensing (PRS) technology and techniques [8,9,10,11,12].

A common PRS approach employs 2-D (2-dimensional) RGB (Red, Green, Blue) imagery, captured with a digital camera for yield estimation; for example [8,9,13]. The 2-D approach can be categorised into two steps: (i) image segmentation (to differentiate the bunch from the background); and (ii) yield estimation, using a suitable bunch metric (i.e., the pixel count of the bunch area in an image). 

Diago et al. [13] used image classification (for segmentation) and a bunch metric to classify ‘background noise’ and ‘grape’ classes. The authors achieved a testing r^2^ of 0.73. An alternative segmentation approach to image classification uses colour thresholding to differentiate grapes from the background. Dunn and Martin [8] presented an RGB colour thresholding approach that applied specific thresholds to the colour properties of an RGB image, generating a binary image—background and grapes. The authors achieved an r^2^ of 0.72. A similar thresholding approach was adopted by Liu, Marden and Whitty [9] and Font et al. [14]. Liu, Marden and Whitty [9] introduced a bunch-level experiment under laboratory conditions with manual colour thresholding for image segmentation, which resulted in a yield estimation r^2^ of 0.77. Additionally, these authors [9] presented a more complex automatic process for image segmentation on the same dataset presented by Dunn and Martin [8], resulting in an improved r^2^ of 0.86. Automatic segmentation stemming from colour thresholding removes the human factor of manual thresholding, resulting in a more robust methodology [15,16]. However, these techniques are more sophisticated than manual thresholding, and therefore require adequate knowledge of computer vision techniques.

The second step, yield estimation, depends on the selected bunch metric. A simple pixel count of the segmented bunches is a favoured metric [8,13], with adaptions to the pixel count presented by Liu, Marden and Whitty [9] and Font et al. [14]. Liu, Marden and Whitty [9] tested five metrics: (i) volume, (ii) pixel count, (iii) perimeter, (iv) berry number and (v) berry size. Yield estimation using the pixel count produced superior results over the remaining metrics [9]. Unlike Liu, Marden and Whitty [9], Nuske et al. [11] avoided image segmentation and used a berry detection algorithm to determine a berry count. The use of the berry count as a yield estimation metric provided an r^2^ of 0.74. Subsequent work on multiple multi-temporal datasets produced r^2^ values between 0.6–0.73 [4]. 

A less common PRS approach utilises an RGB-D (RGB-Depth) camera to capture a 3-D (3-dimensional) model in either mesh or point-cloud format, representing the bunch or vine in a 3-D coordinate system [17]. The use of the Microsoft Kinect™ constitutes an ideal low-cost RGB-D sensor for in-situ imaging of vines [17]. The resulting 3-D models can be used to extract volumetric measurements for yield estimation. A limited number of studies have investigated the utility of the Kinect sensor for volume estimation. For example, Wang and Li [18] and Andújar et al. [19] employed the Kinect sensor under laboratory conditions for the volume estimation of sweet onions and cauliflowers, respectively. To date, the only use of an RGB-D Kinect sensor for yield estimation within viticulture was presented by Marinello et al. [10]. The authors assessed the sensor position for volume estimation of table grape bunches by testing two viewing angles, side-on and bottom-up. Multiple sensor-target distances were tested for the side-on viewing angle. Marinello et al. [10] concluded that a side-on viewing angle with a sensor-target distance of 0.8–1.0 m generated the best results.

An alternative PRS technique combines 2-D imagery with computer vision techniques, whereby 3-D models are created from the 2-D RGB images. Volume estimations are extracted from the subsequent 3-D models, enabling yield estimation calculations. Advanced computer vision techniques allow substantial automation in the process [20,21,22].

RGB and RGB-D technology and techniques incorporated into a suitable methodology present a viable solution for vineyard yield estimation. The established use of RGB imagery is evident, while the novel use of RGB-D imagery shows promise for future yield estimation. However, to date no study has investigated 2-D and 3-D PRS techniques side-by-side for vineyard yield estimation. Examining these two techniques in a commercial vineyard could provide insight into their capabilities and operational potential. 

A key aspect for consideration when implementing these methodologies within a vineyard is the canopy coverage. The combination of essential canopy management practices and the vineyard’s trellis system—particularly a vertical shoot positioned (VSP) system—directly influences canopy coverage, and inevitably the success of PRS techniques for yield estimation. High canopy coverage in the bunch zone results in bunch occlusion from the sensor. The incorporation of a canopy treatment was therefore proposed for this study.

This study aimed to investigate 2-D RGB and 3-D RGB-D PRS techniques for yield estimation in a VSP vineyard, using bunch area/volume estimation. The study was undertaken as three experiments, occurring under laboratory and field conditions. Field conditions were conducted at both bunch- and plant-level. Two canopy treatments were implemented from direct canopy manipulation and were defined as full canopy (FC) and leaf removal (LR). Hypothetically, the LR treatment will produce better estimation results. Furthermore, to achieve the aim of the study, two objectives were determined: to develop independent 2-D and 3-D yield estimation methodologies and to analyse and compare the success of the two PRS techniques for yield estimation.

## 2. Materials and Methods

### 2.1. Study Site

**Figure 1:**
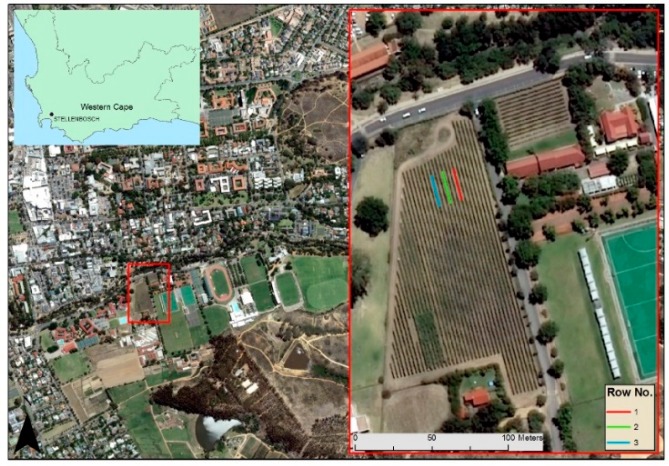
Location of the Shiraz vineyard in Stellenbosch, South Africa. Inset map (red rectangle) shows the three rows used for data collection.

The study was carried out at the end of the 2016/17 growing season in a drip-irrigated Shiraz vineyard at the Welgevallen Experimental Farm located in Stellenbosch, South Africa (33°56′26″ S; 18°51′56″ E). The vineyard was planted in the year 2000, with a grapevine spacing of 2.7 × 1.5 m in a North-South orientation, and lies approximately 157 m above sea level. A seven-wire hedge VSP trellis system with three sets of moveable canopy wires is used in the vineyard. The Stellenbosch area falls within the coastal wine grape region of the Western Cape, which is characterised by a Mediterranean climate with long, dry summers [23]. Thirty-one individual vines were selected across three rows and sampled for this study (Figure 1).

### 2.2. Data Acquisition

Data was acquired between 28 February and 3 March 2017 (harvest), where data collected in situ was implemented with the two canopy treatments. The purpose behind the canopy treatments was to gain a direct line of sight to the bunches, which were generally hidden by the vine’s canopy. No manipulation of the canopy, essentially the normal canopy condition, was classified as the FC treatment. The alternative LR treatment occurs after manual manipulation of the canopy, resulting in complete leaf removal in the bunch zone, effectively displaying the bunches.

**Figure 2:**
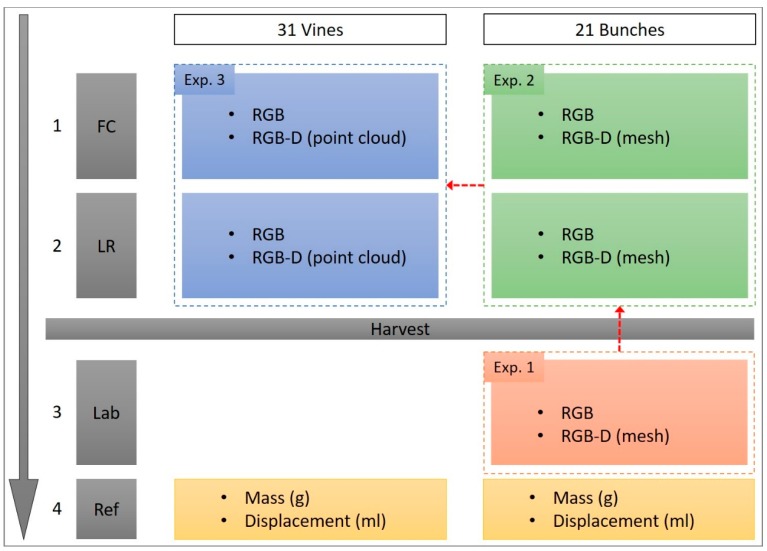
Data-acquisition protocol used in this study. Order of acquisition indicated by the grey arrow. {Key: FC = full canopy; LR = leaf removal; Lab = laboratory; Ref = reference measurements; Exp = experiment}.

Figure 2 illustrates the data-acquisition process that resulted in a total of ten datasets, as outlined by each step:

1. RGB and RGB-D imagery acquired for the FC treatment taken at bunch-level (n = 21; randomly selected and labelled bunches from the 31 vines) and plant-level (n = 31; individual vines). The resulting four datasets included: (i) RGB: bunch; (ii) RGB-D: bunch; (iii) RGB: vine; and (iv) RGB-D: vine.
2. Manual manipulation of the canopy resulted in the environment for the four LR treatment datasets. These datasets were identical to the FC datasets and concluded the in-situ datasets (datasets = 8). At this point, the 31 vines were harvested.
3. RGB and RGB-D imagery of the harvested bunches (n = 21) captured under laboratory conditions resulted in the final two datasets. At this point, all datasets (datasets = 10) have been captured.
4. Reference measurements, captured under laboratory conditions, included mass (g) and displacement (mL) for the individual bunches (n = 21) and individual vines (n = 31).

RGB and RGB-D images were captured by two PRS sensors. A D3200 digital single-lens reflex camera (Nikon, Tokyo, Japan) was used for capturing 24.2-megapixel RGB images. The camera captured images in *auto* mode, with the flash disabled. The second sensor, a Kinect™ V1 (Microsoft, Redmond, WA, USA), was used to capture RGB-D imagery as either a mesh (bunch-level) or a point cloud (plant-level). The following data-acquisition subsections provide experiment-specific details.

#### 2.2.1. Reference Measurements

Reference measurements were collected under laboratory conditions for the 21 individual bunches and the 31 individual vines. Individual bunch mass (g) was recorded with a Mentor scale (Ohaus, Parsippany, NJ, USA), and individual vine mass (g) was recorded with a Viper SW scale (Mettler Toledo, Columbus, OH, USA). Bunch/vine volume measurements were recorded as the displacement (mL) of water when bunches were submerged in a container of water [24]. The mass and volume measurements were used as reference measurements for the estimated measurements derived from the two PRS techniques.

#### 2.2.2. Experiment One: Individual Bunches under Laboratory Conditions

**Figure 3:**
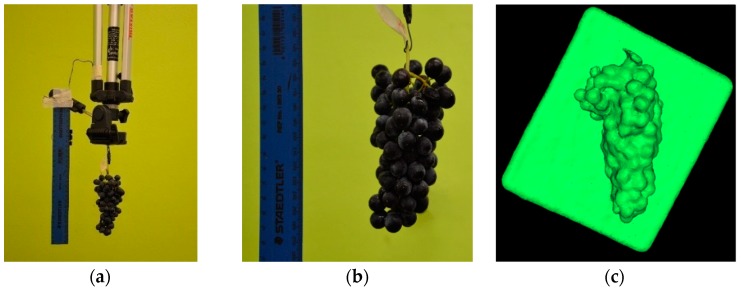
Data acquisition under laboratory conditions. (**a**) Experimental setup for image capture; (**b**) RGB image of an individual bunch with a ruler for reference length; and (**c**) RGB-D (Kinect mesh) of an individual bunch.

The 21 individual bunches were imaged using the RGB camera and Kinect sensor under laboratory conditions; i.e., the laboratory was illuminated with white fluorescent lights and natural light entered through the windows. Each bunch was suspended from a tripod against a green background to maximise image contrast (Figure 3a):

(a) *RGB imagery:* The camera was placed parallel to the suspended bunch at a distance of 60 cm. A single image per bunch was captured for image processing. A ruler was included in each image for length reference (Figure 3b).
(b) *RGB-D (Kinect) imagery:* The Kinect sensor was placed 60 cm proximal to the target and captured individual meshes per bunch, resulting in a solid 3-D model of the bunch. The Kinect sensor, coupled with the Kinect Fusion software (part of Microsoft’s Software Development Kit 1.8 for Windows [25]) running on a laptop, can capture individual meshes. Meshes were captured in *.stl* format with a resolution of 640 voxels/m, and a voxel resolution of 256 × 256 × 256 voxels. A white surface was positioned directly behind the bunch to improve contrast and depth determination, as illustrated by the mesh seen in Figure 3c. During mesh capture, the entire bunch system was rotated, providing different angles of view.

#### 2.2.3. Experiment Two: Individual Bunches in Field Conditions

**Figure 4:**
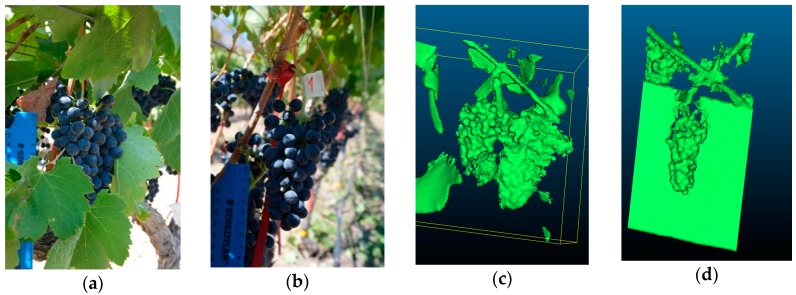
Data acquisition of individual bunches in the field. (**a**) RGB image with full canopy (FC); (**b**) RGB image with leaf removal (LR); (**c**) RGB-D (Kinect mesh) with FC; and (**d**) RGB-D (Kinect mesh) with LR.

Images of the same 21 individual bunches were captured in in-situ conditions under both canopy treatments with the same RGB and RGB-D proximal sensors that were used in experiment one. Here, individual bunches were still attached to the respective vines:

(a) *RGB imagery:* The camera captured images of the individual bunches for both FC (Figure 4a) and LR (Figure 4b) treatments. The camera was positioned approximately 40 cm from the bunch being imaged, maintaining the reference length (ruler) in each image. Image acquisition occurred between 12H00 and 13H00, under natural solar illumination.
(b) *RGB-D (Kinect) imagery:* The same software setup from experiment one was used, which allowed the Kinect to capture an individual mesh per bunch for FC (Figure 4c) and LR (Figure 4d) treatments. The imagery was captured after sunset (approximately 20H00) with artificial illumination. The Kinect was held approximately 60 cm from the bunch and was moved around the bunch axis by hand. For the LR treatment, a board was placed behind the bunches, thereby creating an artificial background to improve volume extraction (Figure 4d).

#### 2.2.4. Experiment Three: Individual Vines in Field Conditions

**Figure 5:**
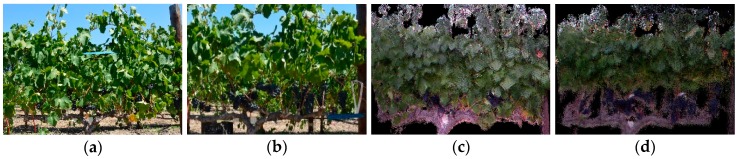
Experiment three data examples at plant-level. RGB imagery of FC (**a**) and LR (**b**) treatments. RGB-D (Kinect point cloud) of FC (**c**) and LR (**d**) treatments.

The same RGB and RGB-D sensors were used to capture in-situ images at plant-level (31 individual vines imaged for both FC and LR treatments):

(a) *RGB imagery:* Imagery was captured at 12H00, under natural solar illumination. The camera was positioned 2 m from the vine, capturing an image for each side of the canopy. This protocol was repeated for both canopy treatments (FC: Figure 5a; LR: Figure 5b).
(b) *RGB-D (Kinect) imagery:* The Kinect sensor captured point clouds—instead of meshes—of the 31 vines, due to the scale difference. Point clouds consisted of thousands of individual points to create 3-D models; for both the FC and LR treatments (Figure 5c,d). The Real-Time Appearance-Based Mapping (RTAB-Map) software [26] was used for point-cloud modelling and regeneration. In RTAB-Map [26], the default filtering parameter and a 3-D cloud decimation (‘thinning’ of the point cloud) value of ‘2’ were used during exportation of the point clouds in *.ply* format. The point cloud was captured per individual row, repeated for both sides of the canopy. The Kinect sensor was hand-held approximately 2 m from the vines at a perpendicular angle and was moved along the row in a north to south direction. The imagery was collected at 19H00, immediately before sunset, using the last natural illumination of the day.

### 2.3. Data Analysis

Data pre-processing and analysis occurred sequentially from experiment one (indicated via red arrows in Figure 2). The canopy treatments existing in experiment two and three had no effect on how the datasets were analysed for yield estimation. The proposed RGB and RGB-D methodologies were created on the LR datasets, before being directly applied to the FC datasets. 

#### 2.3.1. RGB Imagery

RGB images were processed using a custom script in MATLAB^®^ [27], as follows:

1. The reference length (obtained from the ruler) in each image was used to scale the image pixels, creating a calibration value in cm^2^.
2. Manual selection of the region of interest (ROI) containing the relevant bunch/bunches was undertaken. ROIs were strategically digitised so as to capture minimal background.
3. The masked RGB images were then converted to the HSV (hue, saturation, value) colour space and segmented using MATLAB’s [27] *Colour Thresholder* app, part of the *Image Processing Toolbox™*. It was visually evident when selecting the threshold values that the lighting conditions influenced the values. Separate threshold values were therefore determined for the respective experiments. Threshold values were computed using a random training sample from the specific dataset; equivalent to 25% of the experiment’s dataset.
4. After the image segmentation process, the number of segmented pixels was determined (adaption of the pixel count metric [9]) and converted into a pixel area (cm^2^) using the calibration value.

**Figure 6:**
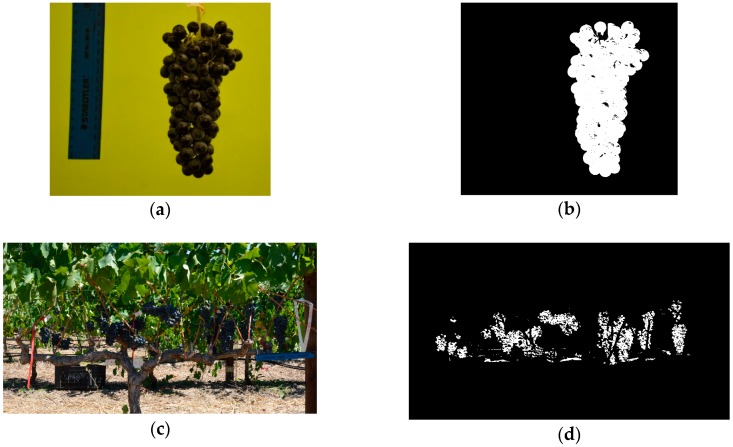
(**a**) Represents the original RGB image, with (**b**) illustrating the segmented binary image at bunch-level. (**c**) An RGB image of an east-facing vine, with (**d**) the segmented binary image at plant-level.

Figure 6 illustrates the image segmentation process for experiment one at bunch-level (Figure 6a,b), and experiment three at plant-level (Figure 6c,d). 

For experiment three, segmentation produced a segmented bunch area image (Figure 6d) for a single side of the vine, with a similar image for the reverse side of the vine. To obtain a single area per vine, the *Total Bunch Area of Vine (TBAV)* was calculated, as follows:

(1) TBAV=(Ae+Aw)/2

where *Ae* was the area of the east-facing side of the vine, and *Aw* represented the vine’s west-facing area. The *TBAV* was in cm^2^. Segmentation success was assessed on the testing subset (25% of the total dataset). A confusion matrix was computed for each experiment, using the predicted values from the segmented binary image versus the actual values of the original RGB image. F1-score and accuracy metrics calculated from the confusion matrix evaluated the segmentation accuracy.

#### 2.3.2. RGB-D (Kinect) Imagery

**Figure 7:**
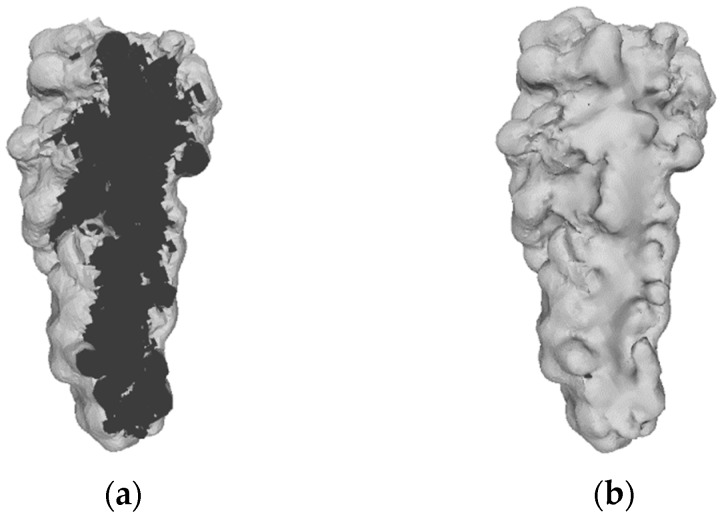
(**a**) Example of mesh prior to reconstruction, and (**b**) the same mesh after Poisson reconstruction.

Due to the two image data types captured—mesh vs. point cloud—RGB-D imagery was processed differently for experiment one and two (mesh: bunch-level), and experiment three (point cloud: plant-level). Data processing for experiments one and two progressed as follows:

1. Each mesh was manually cleaned and sectioned to remove any background, using the *Cross-Section* tool in CloudCompare [28].
2. The cleaned mesh was reconstructed in MeshLab [29], using the *Screened Poisson Surface Reconstruction* [30] method—see Figure 7. Additional cleaning of the mesh was completed ad hoc in MeshLab [29].
3. Volume (cm^3^) of the mesh was calculated in CloudCompare [28].

*Screened Poisson Surface Reconstruction* [30] allows the back of the mesh, which was ‘open’ (Figure 7a), to be reconstructed. It becomes ‘watertight’, as seen in Figure 7b.

The nature of the point-cloud data requires different processing steps to that of the mesh data. This effect was attributed mainly to the necessity of closing the points of the point cloud, thereby producing a ‘watertight mesh’ for volume extraction. 

The point cloud datasets from experiment three were processed as follows:

1. Point clouds were imported into CloudCompare [28], and subsequently cleaned and sectioned to individual vines, focusing on the bunch zone.
2. Bunches were segmented from the point cloud using their colour properties. CloudCompare’s [28] *Filter Points by Value* tool incorporates user-defined thresholds, manipulating the RGB colour space of the point cloud. Threshold values were determined on a random sample (25%) of the LR dataset.
3. A custom-built script in R statistical software [31] was used for calculating the segmented point cloud’s volume, representing the vine’s bunches.

**Figure 8:**
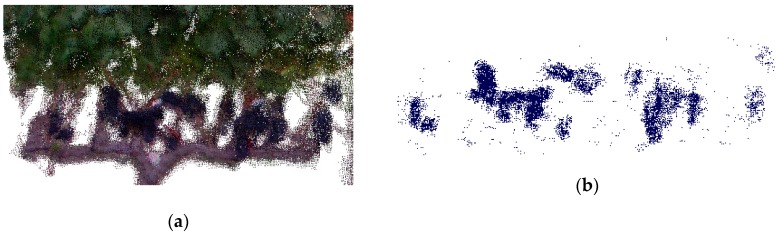
(**a**) The Kinect point cloud for an LR-treated vine (1E—east side) and (**b**) the segmented point cloud of the same vine.

Figure 8 illustrates the raw point cloud (Figure 8a) captured by the Kinect sensor, with the segmented point cloud (Figure 8b) displaying the bunches.

The custom script in R statistical software [31] incorporated the *alphashape3d* package v1.3 [32] to compute the 3-D shape for volume calculation purposes. The *alphashape3d* [32] package includes the *α-shape* algorithm [33] to recover the geometric structure of the 3-D point cloud for volume calculation. The *α-shape* algorithm [33] requires a specific alpha value for computation; hence, the alpha value directly influences the total volume calculated. To adjust an alpha value for our experimental conditions, various levels of alpha were tested on 25% of the dataset and linearly regressed against the reference values. Similar investigative experiments were conducted by Rueda-Ayala et al. [34] and Ribeiro et al. [35] to determine experiment-specific alpha values.

The process described above for experiment three was repeated for both sides of the vine, resulting in two volume measurements per vine. A single volume value per vine was obtained via the *Total Bunch Volume per Vine* (*TBVV*) calculation:

(2) TBVV=(Ve+Vw)/2

where *Ve* was the volume of the vine’s east-facing side, and *Vw* was the volume of the west-facing side. The resultant *TBVV* was in cm^3^ per vine.

#### 2.3.3. Cross-Validation

Five-fold cross-validation was used to develop the yield estimation model for each dataset. Cross-validation was implemented using the *Caret* package [36] in R statistical software [31], repeated ten times for model robustness. The model produced ‘fitted values’, which represented the estimated yield (in grams). Following this, the estimated yield (g) values were linearly regressed against the actual mass (g) to produce a final r^2^ value (coefficient of determination), indicating the potential for yield estimation. The Root Mean Square Error (RMSE) was computed from the linear regression, indicating the yield estimation error (in grams).

## 3. Results

### 3.1. Reference Measurements

Results of the reference measurements indicated a strong relationship between mass and volume at bunch-level (r^2^ = 0.971) and plant-level (r^2^ = 0.996). The established relationships between mass and volume served as the basis for the subsequent experiments, which were used to evaluate the 2-D and 3-D techniques.

### 3.2. Pre-Processing 

The complexity of the RGB-D (Kinect) datasets required two additional pre-processing steps. The first step was the determination of an alpha value required for volume calculation, using the *alphashape3d* package [32]. The second step was volume correction for all Kinect datasets, due to volume estimation errors in the datasets. The segmentation accuracy for the RGB pre-processing has also been included in this section.

#### 3.2.1. RGB Segmentation Accuracy

Experiment one’s segmentation results yielded an F1-score of 0.976, with an accuracy of 0.971. Experiment two resulted in a lower F1-score of 0.842, with an accuracy of 0.781. Lastly, experiment three’s F1-score and accuracy were 0.833 and 0.932, respectively. Solar illumination could contribute to the lowered results presented in the in-situ measurements.

#### 3.2.2. alphashape3d’s Adjusted Alpha Value

**Figure 9:**
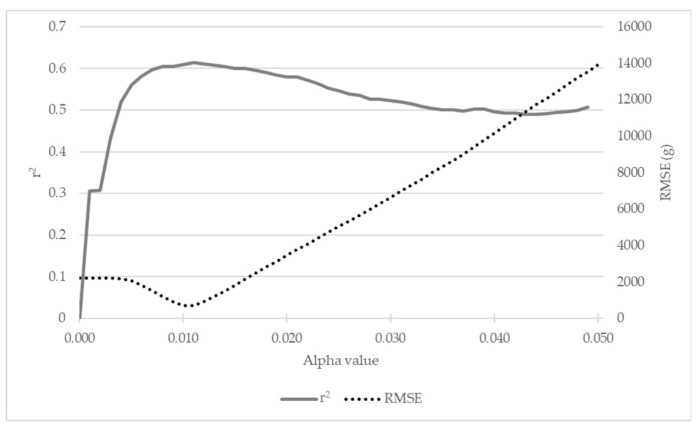
The relevant r^2^ and RMSE values for each alpha value tested for the *alphashape3d* package in the custom R script. Alpha values incremented in 0.001, ranging from 0.001–0.050.

**Figure 10:**
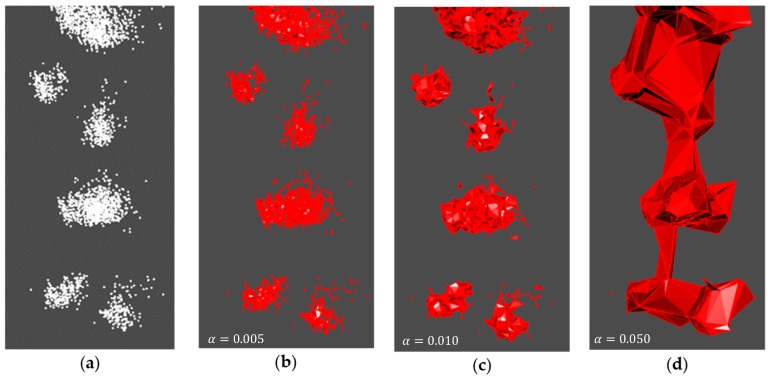
Point-cloud reconstruction testing the alpha value for the *alphashade3d* package. The original point cloud before reconstruction—represented as white points for a visual purpose (**a**), and after reconstruction (**b**–**d**).

Figure 9 represents the r^2^, and RMSE curves for the various alpha values tested. Values tested ranged from 0.001 to 0.050, in increments of 0.001. It is evident from this figure that the alpha value selected (alpha = 0.010) satisfies a high coefficient of determination (r^2^ = 0.605) combined with the lowest RMSE (703.301 cm^3^). Figure 10 provides a visual interpretation of the results from Figure 9. A low alpha value, such as 0.005 (Figure 10b), produced an r^2^ of 0.520 and an RMSE of 2052.751 cm^3^ (sample dataset mean volume = 1961.875 cm^3^). Conversely, a high alpha value of 0.050 (Figure 10d) produced an r^2^ of 0.506 and an RMSE of 13 936.31 cm^3^. The selected alpha value of 0.010 (Figure 10c) was subsequently used for all further analyses.

#### 3.2.3. Kinect Volume Correction

**Figure 11:**
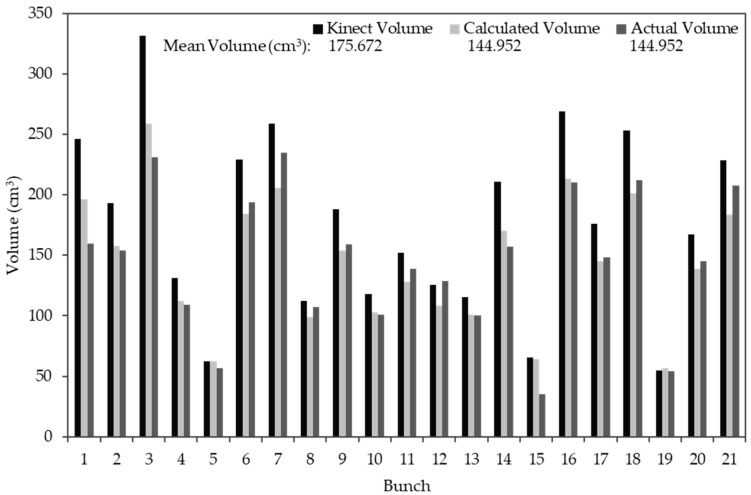
Experiment one’s results, for the 21 individual bunches, illustrating the volume estimation error by the Kinect sensor.

Figure 11 shows a volume estimation error present in experiment one’s data, which aligns with a subsequent review of the literature [10,18,19]. The 21 bunches have a mean actual volume (the reference volume) of 144.952 cm^3^ and a mean estimated Kinect volume of 175.672 cm^3^. Overestimation by the Kinect V1 sensor was evident from the results. Volume correction via cross-validation was therefore subsequently incorporated into the methodology, where a mean corrected volume of 144.952 cm^3^ was achieved. Thereafter, the correction was applied to the remaining Kinect datasets.

### 3.3. RGB Results

**Figure 12:**
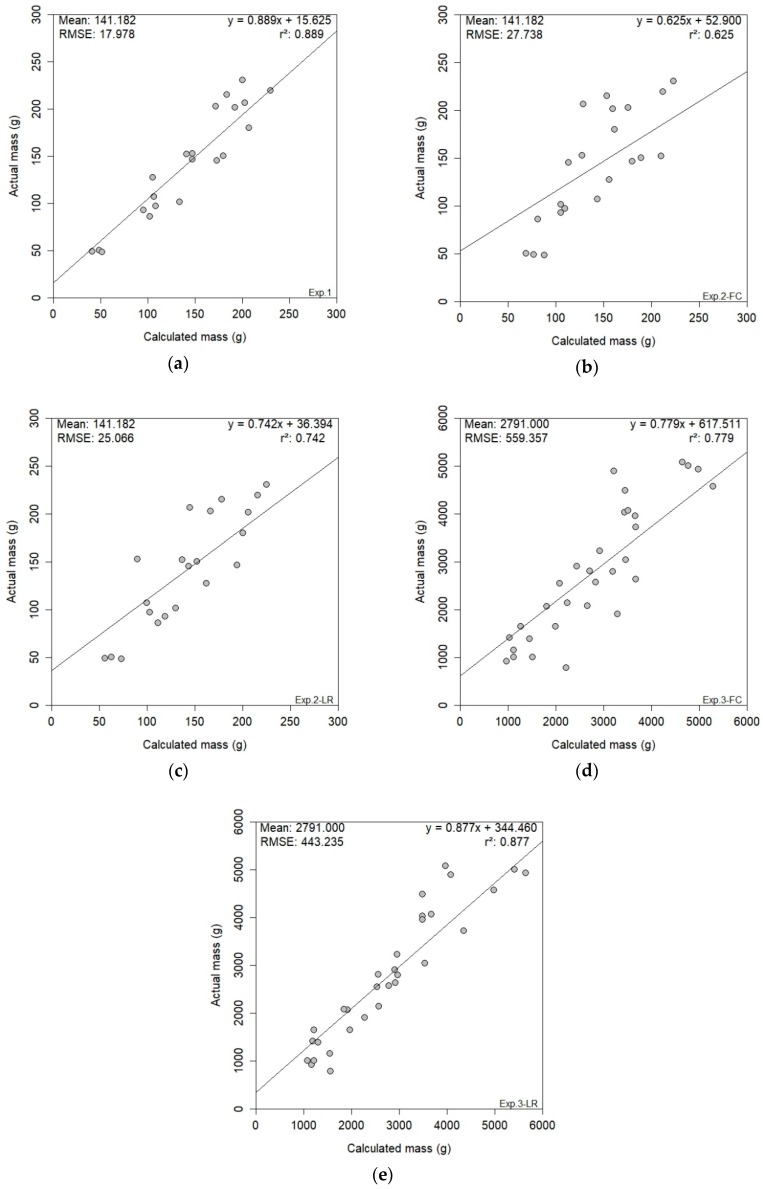
RGB results presented for the three experiments; experiment one (**a**), experiment two FC (**b**) & LR (**c**) and experiment three FC (**d**) & LR (**e**). *{Key: Exp. = experiment; FC = full canopy; LR = leaf removal}.*

Figure 12 shows the results for the three experiments that used 2-D RGB digital imagery. The best results were obtained in experiment one (Figure 12a), which produced an r^2^ of 0.889 and an RMSE of 17.978 g. The level of accuracy achieved in experiment one can be attributed to the controlled laboratory conditions and supports the proposed methodology for yield estimation. Applying this methodology to in-situ bunches produced less accurate results, as seen in experiment two. Experiment two’s FC treatment (Figure 12b) produced the lowest yield estimation results for 2-D RGB imagery, with an r^2^ of 0.625 and an RMSE of 27.738 g. The LR treatment’s (Figure 12c) r^2^ and RMSE values were 0.742 and 25.066 g, respectively. The lesser FC values were directly attributed to the canopy coverage present, as the bunches were partially occluded from the sensor’s view. The effect of the canopy treatment was evident when comparing the results. At plant-level (experiment three), the same pattern was present between the canopy treatments. The FC (Figure 12d) treatment of experiment three produced an r^2^ of 0.779, while the LR (Figure 12e) treatment produced an even higher r^2^ of 0.877. The respective RMSE values were 559.357 g and 443.235 g. The success of yield estimation in experiment three, specifically the LR treatment, supports the methodology’s capability for 2-D RGB yield estimation. The in-situ yield estimation was preferable at plant-level, which may be attributed to the lighting conditions and the success of the colour thresholding for bunch segmentation at plant-level.

### 3.4. RGB-D Results

**Figure 13:**
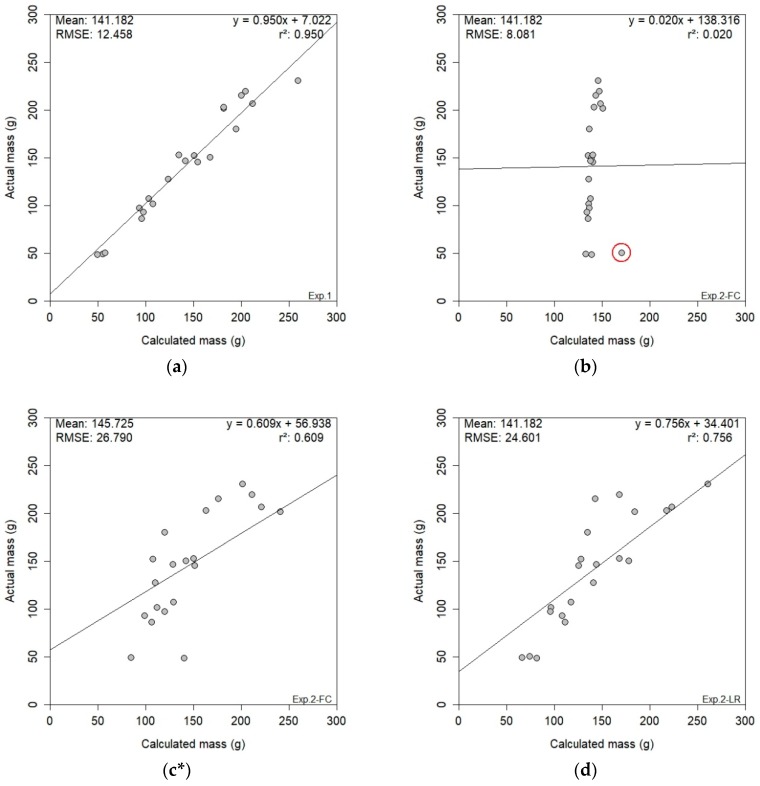

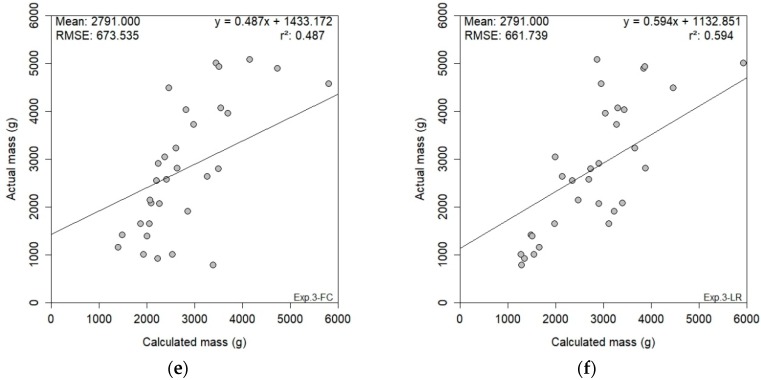
Presented RGB-D results of the three experiments; experiment one (**a**), experiment two FC (n = 21) (**b**), experiment two FC* (n = 20) (**c**), experiment two LR (**d**) and experiment three FC (**e**) & LR (**f**). {Key: Exp. = experiment; FC = full canopy; LR = leaf removal; *statistical outlier removed, resulting in 20 bunches}.

Figure 13 illustrates the RGB-D results obtained for the three experiments. The unrivalled results obtained in experiment one (Figure 13a) produced an r^2^ of 0.950 and an RMSE of 12.458 g—the best-performing results presented in this study. The Kinect sensor favoured the controlled conditions of the laboratory, specifically the artificial illumination as a source of light. Applying the same methodology to experiment two (in-situ bunches) resulted in a lower yield estimation performance for both canopy treatments. The FC treatment (Figure 13b) produced an abnormally low r^2^ of 0.020, with an RMSE of 8.081 g. A statistical outlier in the data was evident when the results were analysed. With the removal of this outlier, the modified FC results (n = 20) improved drastically, with a new r^2^ of 0.609 and an RMSE of 26.790 g (Figure 13c). In contrast, the LR treatment (Figure 13d) resulted in an r^2^ of 0.756 and an RMSE of 24.601 g, which aligned with the LR results for bunch-level obtained with RGB imagery (Figure 12c). At plant-level, the unfavourable results of the FC treatment (Figure 13e) generated an r^2^ of 0.487 and an RMSE of 673.535 g. However, the LR treatment (Figure 13f) provided some promise for the Kinect sensor at plant-level, achieving an r^2^ of 0.594 and an RMSE of 661.739 g. The same effect of the canopy treatment was evident in the RGB-D results as in the RGB results, with the LR treatment producing a better yield estimation agreement. The results of experiment three indicated a limitation within the proposed methodology for RGB-D yield estimation at plant-level. Such limitation could be from the data-acquisition process, or the image segmentation within the data analysis.

**Figure 14:**
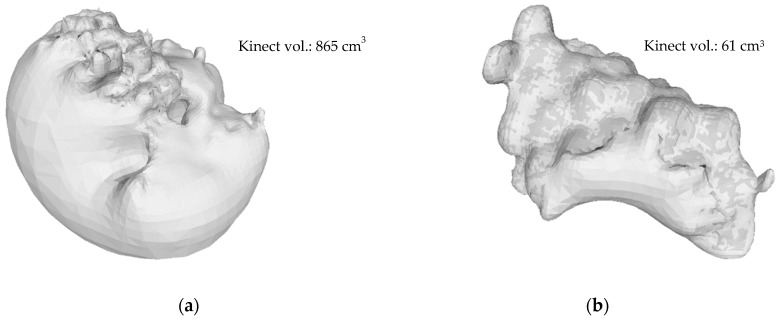
Illustration of *Screened Poisson Surface Reconstruction*. The reconstructed bunch (circled in red—Figure 13b) with the incorrect volume (**a**), and an example of a reconstructed bunch of the correct volume (**b**).

The poor results obtained in experiment two’s FC dataset can be attributed to the mesh reconstruction step in the data-analysis process. Exaggerated reconstruction of bunches presents a potential limitation of the *Screened Poisson Surface Reconstruction* algorithm [30], as depicted in Figure 14. The statistically-outlying bunch circled in red (Figure 13b) produced a Kinect volume of 856 cm^3^, as illustrated in Figure 14a, when the actual volume was only 35 cm^3^. An example of an accurately reconstructed mesh (Figure 14b) was included for visual comparison. This anomaly was unavoidable during data processing.

## 4. Discussion

To date, most studies have employed 2-D RGB imagery for vineyard yield estimation at both bunch- and plant-level. However, only Marinello et al. [9] have investigated the use of 3-D RGB-D (specifically making use of a Kinect sensor) imaging for vineyard yield estimation. The research we have presented investigated 2-D and 3-D PRS techniques for vineyard yield estimation. The study was undertaken as three experiments, consisting of bunch-level and plant-level datasets, with in-situ measurements captured for the two canopy treatments (FC and LR). The following subsections discuss the results of the two PRS techniques in further detail. 

### 4.1. Using 2-D RGB Imagery for Yield Estimation

The presented results using RGB imagery for yield estimation are robust and support the 2-D PRS technique. Experiment one (r^2^ = 0.889; RMSE = 17.978 g) illustrated the success of RGB imagery in a controlled environment for yield estimation at bunch-level. At plant-level, similar results were presented for the LR treatment in experiment three (r^2^ = 0.877; RMSE = 443.235 g). The success of the methodology under both laboratory and field conditions supports the use of colour thresholding for image segmentation, and the adapted pixel area metric for yield estimation.

Colour thresholding for image segmentation was favoured by several studies [8,9,14]. In our study, the use of the HSV colour space for thresholding has proven fruitful. The HSV colour space is supported by Font et al. [14], who achieved favourable results (estimation error of 13.55%) when working with the H layer for segmentation purposes. At bunch-level, experiment one’s result (r^2^ = 0.889) shows an improvement to the result (r^2^ = 0.77) presented by Liu, Marden and Whitty [9]; similarly conducted under laboratory conditions. This aspect was a noteworthy improvement, especially considering the manual nature of the colour thresholding. At plant-level, the manually produced results of experiment three (FC: r^2^ = 0.779; LR: r^2^ = 0.877) align with the automated classification results (r^2^ = 0.865) of Liu, Marden and Whitty [9]. Here, Liu, Marden and Whitty [9] use the same 1×1 m image dataset as Dunn and Martin [8], as opposed to the plant-level imagery used in our study. Additionally, the colour thresholding approach of our study outperformed the image classification approach (test r^2^ = 0.73) [13] to segmentation. 

We presented an adaption of the pixel count metric for yield estimation, which expands on current literature [8,9,13,14]. Of the five metrics tested by Liu, Marden and Whitty [9], the pixel count produced the best results. We found the incorporation of a calibration length (the ruler) for pixel count resulted in an improved quantitative pixel area (cm^2^) for yield estimation. Again, our results at bunch-level (r^2^ = 0.889) improved the bunch-level results (r^2^ = 0.77) conducted in laboratory conditions—as presented by Liu, Marden and Whitty [9], who were specifically testing the various yield estimation metrics. At plant-level, our LR treatment (r^2^ = 0.877) outperformed all current literature to date, with our FC treatment (r^2^ = 0.779) representing a slight improvement for in-situ measurements. The presented pixel area (cm^2^) metric also improved on the berry count results [4,11], with the highest berry count r^2^ (0.74) presented still being lower than our plant-level pixel area r^2^ (0.779) for the FC treatment. However, the potential of the berry count is applicable across all cultivars, as it does not depend on the colour of the berry [11]. 

The limitation of slight distance variations between the camera and bunches within each image was resolved by the incorporation of the reference length (the ruler). The necessity of determining the calibration length for each image outweighs the added processing requirement of this step. Overall, this allows improved yield estimation success, as represented in our results. Future work could attempt to automate this process. However, a more restrictive limitation was the human involvement in determining the appropriate threshold values; this could explain the lowered in-situ estimation performance at bunch-level. Future work could investigate a more automated methodology, thus alleviating this limitation.

### 4.2. Using 3-D RGB-D Imagery for Yield Estimation

To date, the work presented by Marinello et al. [10] is the only literature supporting the use of the Kinect V1 sensor for vineyard yield estimation. Marinello et al. [10] used table grape bunches to determine the optimal viewing angle and distance for the sensor. The authors concluded that a side-on view of the bunch, with a distance of between 0.8–1.0 m, produced the least variability in mass estimations. The current findings of our study, which incorporated RGB-D imagery for yield estimation across the three experiments, are therefore the most comprehensive findings to date. The presented study exemplifies the potential of 3-D PRS techniques for yield estimation, specifically a cost-effective sensor like the Kinect V1.

The nature of our study’s datasets resulted in separate data analysis between the bunch- and plant-level datasets. Astounding results were obtained in experiment one (r^2^ = 0.950; RMSE = 12.458 g). It was evident that the Kinect sensor favours ideal laboratory conditions, allowing accurate yield estimation at bunch-level. The ramification of less-favourable conditions, such as in-situ monitoring, became apparent in experiment two (bunch-level) and three (plant-level).

Although no bunch-level studies have used a Kinect sensor for vineyard yield estimation, a similar approach under laboratory conditions, estimating the volume of cauliflowers, was presented by Andujar et al. [19]. The authors were able to achieve an r^2^ of 0.868 when regressing the estimated volume against the known fruit mass. Conversely, our methodology enables the relationship between fruit volume and mass to be modelled, allowing the use of the adjusted mass (calculated from the model) for subsequent yield estimation. A fundamental difference between our methodology and that of Andujar et al. [19] is the method in which the 3-D model was captured. Our methodology captured a 3-D mesh of the fruit, while Andujar et al. [19] captured a 3-D point cloud of the vegetable. This required model reconstruction, constructively producing a ‘watertight mesh’. Our improved results at this level (r^2^ = 0.950) can be accredited to this fundamental difference. Interestingly, both methodologies used the same 3-D reconstruction method—*Screened Poisson Surface Reconstruction* [30] found in MeshLab [29]. 

The primary limitation of the proposed methodology at bunch-level was a combination of dataset quality and the *Screened Poisson Surface Reconstruction* method [30]. The consequence of a poor-quality mesh became apparent when bunch reconstruction resulted in significant defects, as seen in Figure 14a. Such imperfections directly affect the potential for accurate yield estimation, exemplified by the results of experiment two’s FC dataset (r^2^ = 0.020; in Figure 13b). Future research is necessary to gain a better understanding of this shortfall in the methodology.

This study presented the novel use of a Kinect RGB-D sensor for in-situ vineyard yield estimation at plant-level (experiment three). The presented plant-level methodology produced promising results for the LR treatment (r^2^ = 0.594; RMSE = 661.739 g), while the effect of the canopy coverage was evident in the FC treatment (r^2^ = 0.487; RMSE = 673.535 g). Future work should improve on the presented methodology, potentially overcoming several limitations. Additionally, the nature of the Kinect V1 sensor results in the fundamental constraint of being suited to indoor use only, as solar illumination produces excessive interference in the captured imagery [19]. Future work should make use of the Kinect V2 sensor, since several improvements to the sensor have been implemented, effectively allowing improved outdoor imagery to be captured [37]. RGB-D sensors, specifically the Kinect V2, are being incorporated in terrestrial vehicles as cheap sensor alternatives for vineyard modelling and yield estimation, which is demonstrated by [38].

### 4.3. The Operational Potential of Developed Methodologies

Both the presented 2-D RGB and 3-D RGB-D methodologies achieved acceptable accuracies across the three experiments. Our results (Figure 12 and Figure 13) support the use of PRS technology and techniques for vineyard yield estimation, especially for VSP-trained Shiraz vineyards. The nature of the presented work was conceptualised to assess 2-D and 3-D PRS sensors side-by-side, a novelty in the vineyard yield estimation domain. 

Experiment one illustrated the capability of these two techniques for successful yield estimation of individual bunches, where the Kinect RGB-D sensor (r^2^ = 0.950) outperformed the digital RGB sensor (r^2^ = 0.889). The suitability of the lighting under laboratory conditions coupled with the Kinect’s ability to capture a 3-D model of the bunch both contribute to the success of the Kinect sensor over the RGB sensor. Nonetheless, robust methodologies were established in a controlled environment.

Experiment two tested the established methodologies in situ, under both FC and LR canopy treatments. The produced results of experiment two—and experiment three—confirmed the hypothesis of a superior yield estimation agreement under the LR canopy treatment. If good canopy management practices are established early in the season, better yield estimation results than those obtained under the FC treatment could be achieved. Both sensors produced similar results for FC [approximate r^2^ = 0.61; using Kinect’s modified results (n = 20)] and LR (approximate r^2^ = 0.75) treatments in experiment two. 

The success of the two PRS methodologies can be differentiated at plant-level by the results of experiment three. Unlike experiment one, the RGB methodology outperformed the RGB-D methodology for yield estimation. The RGB results (r^2^ = 0.877) significantly outclassed the RGB-D results (r^2^ = 0.487) under the FC treatment, whereas a smaller margin between the RGB (r^2^ = 0.779) and RGB-D (r^2^ = 0.594) sensors occurred under the LR treatment. Inference behind the differing results can lead to the following observations. The different lighting conditions influenced the results: the RGB imagery was collected at midday, while the RGB-D imagery was collected immediately before sunset. Additionally, the continuous movement of the Kinect sensor (RGB imagery captured in a stationary position) could have contributed to lowered RGB-D results. Further research is encouraged, using a standardised experimental setup, where feasible, to create more favourable conditions under which both sensors can operate. 

For the RGB and RGB-D datasets, the LR treatment yielded higher estimation agreements compared with the FC treatments. The LR treatment was adopted to create an ideal in-situ environment for yield estimation. The commercial feasibility of complete leaf removal in the bunch zone is not practical in some viticulture regions, since there are adverse effects associated with this practice (such as ‘sunburn’). However, this practice can be implemented in specific zones of the vineyards, enabling an approximation of the total yield using a monitoring strategy. The FC results provide a better indication of the commercial readiness of the developed methodologies. Future work should determine an optimal canopy coverage that favours both sensor operationality and commercial farming methods.

Overall, the use of the Kinect sensor as a cost-effective RGB-D sensor for vineyard yield estimation, specifically at bunch-level, is supported by the results obtained for experiment one. However, the robustness of the RGB methodology is evident across all three experiments, with substantial plant-level results obtained in situ. A better understanding of the current limitations would allow further improvements to the methodologies. To this end, the results obtained in this study support the potential for operationalisation of both PRS sensors. A refined methodology could see commercially favourable data-acquisition methods implemented on a larger dataset, with fully automated data processing. See for example [15,16]. The commercialisation of such methodologies could become more feasible, due to the simplicity and robustness of the current methodologies, coupled with improved yield estimation accuracy.

## 5. Conclusions

A novel approach to a side-by-side investigation of 2-D and 3-D PRS techniques for successful vineyard yield estimation has been presented. This study assessed RGB imagery captured by a digital camera and RGB-D imagery captured by a Kinect V1 sensor across three experiments, with in-situ measurements obtained under two canopy treatments. Our results show that the Kinect RGB-D sensor produced the highest yield estimation agreement under laboratory conditions (bunch-level). At bunch-level, RGB and RGB-D PRS techniques performed equally under both canopy treatments for in-situ yield estimation. At plant-level, the best in-situ results were obtained using the RGB imagery, which significantly outperformed the RGB-D results. Both sensors support the use of PRS technology and techniques for vineyard yield estimation, with improved accuracies presented. The results of this study confirm the operational potential of 2-D RGB imagery for accurate yield estimation, with the recommendation that future work should investigate a more automated RGB methodology suitable for operational environments. Regarding the presented RGB-D methodology, the Kinect demonstrates the potential for vineyard yield estimation using 3-D RGB-D imagery. Future work should investigate the use of the Kinect V2 sensor coupled with suitable lighting conditions for in-situ yield estimation.
